# Editorial: Intra and inter organ cross-talk and cellular communication

**DOI:** 10.3389/fendo.2023.1209436

**Published:** 2023-05-12

**Authors:** Pierre-Damien Denechaud, Nabil Rabhi

**Affiliations:** ^1^ Institut des Maladies Métaboliques et Cardiovasculaires, UMR1297, Inserm, Université Toulouse 3, Toulouse, France; ^2^ Department of Biochemistry & Cell Biology, Boston University School of Medicine, Boston, MA, United States

**Keywords:** obesity, cancer, signaling molecules, metabolism & obesity, cell communication, organ cross talk

Organ crosstalk and organs intracellular communications are the foundation for maintaining whole-body metabolic homeostasis. This constant dialogue at the organ and cellular levels is key to adapting to environmental fluctuations, assessing energy supplies, and, to a larger extent, organisms surviving. Theoretically, any molecule that cells can sense could be leveraged as a communication tool creating illimited possibilities to fine-tune the message and thereby the specificity of cellular repose. Indeed, signaling molecules including growth hormones, chemokines, peptides, and metabolites are used as means to remotely inform the whole-body state and dictate crucial decisions such as growth, survival, or programmed death.

A growing body of evidence indicates that chronic overnutrition promoting disruption of these interactions leads to pathological conditions such as obesity, type 2 diabetes, liver diseases, cardiovascular diseases, and cancer. Importantly, those interactions can be hijacked by cancer cells to support their expansion or survival.

The purpose of this Research Topic is to highlight new advances in this area and to help understand how metabolic status and metabolic diseases influence inter-organ crosstalk and communication.

Obesity is a highly prevalent chronic disease characterized by excessive fat accumulation. It is the body’s response to environmental stimuli and genetic predisposition. In recent years, the gut microbiota has gained significant attention as a major environmental factor for energy homeostasis maintenance and host immunity. In this Research Topic, Wu et al. summarize the current knowledge on the role of gut microbiota in regulating whole-body metabolic homeostasis through a close cross-talk with adipose tissues. The authors, systematically discuss recent evidence demonstrating the central role of gut microbiota in influencing adipose tissue physiology and the development of metabolic disease and obesity. Interestingly, this relationship is a two-way communication system where adipocytes can act *via* adipokines to modulate the gut microbiota. These findings are particularly relevant as they can provide potential intervention strategies for obesity treatment.

Chronic low-grade inflammation is another hallmark of obesity and metabolic diseases. In this Research Topic, Somm and Jornayvaz summarize the emerging roles of interleukin-18 (IL-18), an interesting pleiotropic cytokine, that has been recently shown to control energy homeostasis. Indeed, IL-18 role in host defense against infections and regulation of the innate and adaptative immune response are well known. However, the development of animal model transgenic mouse models for IL-18 revealed an expected involvement in metabolism and obesity development. The authors discuss a body of work uncovering the physiological role of IL-18 in regulating metabolic tissue in mouse models and interesting clinical observations suggesting its potential role in human metabolic diseases including obesity, diabetes, and non-alcoholic fatty liver. Finally, the authors present their perspective of burning questions that need answering to fully understand the metabolic role of IL-18.

While secreted factors are important players in inter-organ communications the transcriptional regulation of their production and secretion by intracellular factors is crucial to maintain metabolic homeostasis. Carbinatti et al. tackle this aspect by reviewing new insights into the inter-organ crosstalk mediated by glucose sensor transcription factor ChREBP (Carbohydrate Responsive Element Binding Protein). This unique transcription factor originally recognized for its central role in controlling of glycolysis and lipogenesis has recently emerged as a key regulator of secreted factors in multiple organs. The authors summarize novel finding implicating ChREBP in the control of hepatokine and metabolite production to ensure an adequate metabolic adaptation and discuss how alteration in its expression drives pathological conditions.

Over the past decade, extensive clinical and experimental evidence linking obesity to the progression of multiple cancer subtypes had emerged. Indeed, the microenvironment during obesity seems to be suitable for cancer cells to thrive and develop. In an extensive review, Ennis et al. highlight the crosstalk between the breast tumor and its environment most notably the establishment of close intercellular communication between tumor cells and adjacent adipocytes. The authors outline the current state of knowledge highlighting how the microenvironment created by type 2 diabetes predisposes to a more aggressive tumor. Those characteristics are particularly important since they are not considered in the treatment plans for breast cancer patients with type 2 diabetes. In this same topic, Aird et al. demonstrate an important communication axis between cancer cells and adipocytes. Interestingly, the authors show that hypoxia in both breast and ovarian cancer cells alters adipocyte metabolism (metabolites and lipolysis) to support cancer cell metabolism.

In summary, the present Research Topic brought together perspectives from different angles, which we believe will help to emphasize the importance of inter-organ crosstalk in maintaining metabolic homeostasis. Although important, those communications axes represent a significant vulnerability that is closely linked to metabolic dysfunction and could be used by cancer cells to fuel their survival and aggressiveness. Importantly, a better understanding of intra and inter-organ cross-talk and cellular communication in physiology and disease will be key for innovative treatments against multiorgan diseases such as obesity and type 2 diabetes.

## Author contributions

P-DD wrote the Editorial and NR edited it. All authors contributed to the article and approved the submitted version.

